# An evaluation of data processing when using the ActiGraph GT3X accelerometer in non‐ambulant children and adolescents with cerebral palsy

**DOI:** 10.1111/cpf.12795

**Published:** 2022-11-23

**Authors:** Trille Jakobsson, Katarina Lauruschkus, Åsa B. Tornberg

**Affiliations:** ^1^ Department of Health Science, Faculty of Medicine Lund University Lund Sweden

**Keywords:** accelerometer, cerebral palsy, children and adolescents, non‐ambulant

## Abstract

**Purpose:**

To evaluate vertical acceleration, vector magnitude, non‐wear time, valid day classifications, and valid period classifications in the data processing phase when using the ActiGraph GT3X accelerometer in non‐ambulant children and adolescents with cerebral palsy (CP).

**Material and Methods:**

Accelerometer data retrieved from 33 non‐ambulant children and adolescents (4–17 years) with CP were analysed. Comparisons of (i) vertical acceleration versus vector magnitude, (ii) two different non‐wear times, (iii) three different settings to classify a day as valid and (iv) two different settings to classify a period as valid were made.

**Results and Conclusions:**

Vector magnitude and a non‐wear time of at least 90 consecutive minutes statistically significantly increased minutes recorded per day, especially for sedentary time. There was a statistically significant difference in numbers of valid days depending on time criteria set to determine a valid day, whereas there was no statistically significant difference in valid periods using 3 compared to 4 days. This study suggests using the pre‐settings in ActiLife; vector magnitude, non‐wear time of 90 consecutive minutes, 500 min recorded per day with periods of at least 3 valid days when assessing physical activity objectively by the ActiGraph GT3X accelerometer in non‐ambulant children and adolescents with CP.

## 1 INTRODUCTION

Cerebral palsy (CP) is the most common physical disability among children and adolescents globally and refers to a group of disorders in the development of motor control and posture which are caused by a brain injury that occurred before the age of 2 years (Rosenbaum et al., 2007). One way of describing the varying severity of impairment in children and adolescents with CP is to use the five‐level Gross Motor Function Classification System Expanded and Revised (GMFCS‐E&R) (Palisano et al., 1997). Children and adolescents with CP, GMFCS‐E&R levels IV and V are non‐ambulant and unable to sit or walk without support, and hence in need of assistance to be physically active (Palisano et al., 1997).

To understand how physically active non‐ambulant children and adolescents with CP are, one needs to assess their physical activity (PA). PA is any physical movement produced by skeletal muscles that require energy expenditure (Caspersen et al., 1985). Sedentary behaviours (SED), on the other hand, are any waking behaviours that involve minimal physical movement, such as sitting or lying, and require an energy expenditure of less than 1.5 metabolic equivalents of task (Tremblay et al., 2017). There are various methods available to assess PA in children and adolescents. One of them is accelerometry—a method that monitors PA objectivity. The use of accelerometers to monitor PA and SED has rapidly increased over the last years in children and adolescents, regardless of whether they live with CP or not (Adamo et al., 2009; Sirard & Pate, 2001; With et al., 2016). Currently, some of the most common accelerometers used in research are those made by ActiGraph (Migueles et al., 2017).

ActiGraph accelerometers obtain accelerations from movement of the body part to which the accelerometer is attached. Through various algorithms, raw accelerometer data are transformed into activity counts (Arvidsson et al., 2019). The number of activity counts accumulated in a specific time interval determines the intensity of PA or SED, with a higher activity count being accumulated representing a higher intensity of PA or SED. PA is often divided into light‐intensity PA (LPA) and moderate‐to‐vigorous PA (MVPA), with LPA being classified as PA between 1.5 and 3.0 metabolic equivalents of task and MVPA ≥ 3.0 metabolic equivalents of task (Mendes et al., 2018).

The number of activity counts accumulated from raw accelerometer data may be altered by choosing activity counts accumulated from only the vertical axis; unilateral acceleration (VA) or by three axes, the vertical, medio‐lateral and antero‐posterior axes; triaxial acceleration converted into a vector magnitude (VM) (Arvidsson et al., 2019; Migueles et al., 2017).

Other than choosing VA or VM when assessing SED, LPA and MVPA from Actigraph accelerometers, cut‐points, non‐wear time and how to define a valid day and a valid period are also required to be able to determine PA levels in the data processing phase, that is, after data collection (Arvidsson et al., 2019; Migueles et al., 2017).

Cut‐points are intensity thresholds for identifying SED, LPA and MVPA (Cain et al., 2013; Evenson et al., 2008; Freedson et al., 1998). Non‐wear time on the other hand is defined as a consecutive period of zero activity counts accumulated, therefore considered as a period when the accelerometer is not worn and thus this period is removed from further analysis in the data processing phase (Kirkwood & Sterne, 2003; Pallant, 2020). A valid day is a day meeting the set number of minutes needed to be recorded per day, with a valid period being a set number of days meeting the specified number of minutes (Arvidsson et al., 2019; Migueles et al., 2017).

For non‐ambulant children and adolescents with CP there is no consensus regarding choice of VA or VM, what cut‐points to use, non‐wear time to set and how to define a valid period in the data processing phase, potentially affecting the final amount of SED, LPA and MVPA being retrieved and able to be analysed.

Therefore, this study aimed to evaluate settings made in the data processing phase during assessment of PA by accelerometery in non‐ambulant children and adolescents with CP. Comparisons of (i) VA versus VM, (ii) 60 versus 90 consecutive minutes of non‐wear time (iii) 300, 500 or 600 min or more recorded per day to classify a day as valid and (iv) 3 versus 4 days of either at least 300, 500 or 600 min recorded per day to classify a period as valid were made.

## 2 METHOD

### 2.1 Population

Accelerometer data were drawn from participants in two randomized clinical trials conducted in Skåne, Sweden, between 2017 and 2020 (Romanzini et al., 2014; Trost et al., 2016). Non‐ambulant children and adolescents with CP who were enrolled at Child and Youth Habilitation Services in the Skåne Region, Sweden, were eligible. In total, 33 children and adolescents were recruited between the ages of 4 and 17 years (mean 12.36 ± 3.43). Sixteen of the participants were female, and all participants had before the studies been classified as having physical impairments corresponding to a GMCFS level IV or V by a paediatric physician or physiotherapist at their habilitation centre. Nineteen children and adolescents had CP, GMCFS level IV and 14 had CP GMCFS level V. Before the studies, the participants had also been classified with either spastic or dyskinetic CP by a paediatric physician, of which 25 had spastic CP and 8 had dyskinetic CP (Romanzini et al., 2014; Trost et al., 2016).

The two randomized clinical trials were approved by the Regional Ethics Committee in Lund, Sweden (EPN‐dnr 2017/67 and EPM‐dnr 2019‐00106) and are registered at ISRCTN (ISRCTN10569363) and ClinicalTrial.gov (LU2019LEER).

### 2.2 Data collection

An ActiGraph GT3X accelerometer (ActiGraph LLC) was used to collect activity counts. The accelerometer collected activity counts from three axes—the vertical, medio‐lateral and antero‐posterior axes (Migueles et al., 2017; Oftedal et al., 2014)—making it possible to retrieve both VM and VA in ActiLife software version 6.13.4 (ActiGraph LLC). The accelerometer was placed above the iliac crest in line with current recommendation of accelerometer device placement in paediatric populations (Aadland et al., 2018; Evenson et al., 2008; Migueles et al., 2017). Before testing, sampling frequencies were set to 30 Hz (Migueles et al., 2017) and time intervals (epoch length) to categorize activity counts accumulated were set to 10 s (Choi et al., 2011; Migueles et al., 2017).

The accelerometer was worn for up to four time‐periods of seven consecutive days over a year for every child or adolescent. The child or adolescent was asked to wear the accelerometer all waking hours, except for when the device might be exposed to an excessive amount of water such as when the child or adolescent was performing water exercise or showering (Romanzini et al., 2014; Trost et al., 2016).

### 2.3 Data processing

ActiLife software version 6.13.4 (ActiGraph LLC) was used to accumulate SED, LPA and MVPA from activity counts retrieved from the ActiGraph GT3X accelerometer. Evenson et al.'s cut‐points were used classifying activity counts as SED ≤ 100 activity counts per minute (≤16.67 per 10 s), LPA as 101–2295 activity counts per minute (16.83–382.5 per 10 s) and MVPA as ≥2296 activity counts per minute (≥382.67 per 10 s) (Evenson et al., 2008). Non‐wear time was excluded from the analysis and set as either 60 consecutive minutes of non‐wear time or 90 consecutive minutes.

A filter was set to obtain accelerometer data from only waking time, that is, from 06:00 AM to 23:59 PM for all days. Further, a low‐frequency filter was applied to detect minor movements more specifically (Cain et al., 2013).

This resulted in four data sets retrieved from ActiLife (Table 1). Each data set showed all days with monitored data. Total minutes per day and total time in SED, LPA and MVPA were given, depending on which settings in VA versus VM and non‐wear time that had been chosen. Days were lined up to clearly demonstrate which days belonged to the same consecutive period of 7 days.

**Table 1:** Data sets retrieved from ActiLife^a^

VA60:	Days with SED, LPA and MVPA accumulated from VA and with a non‐wear time of at least 60 consecutive minutes of zero counts
VA90:	Days with SED, LPA and MVPA accumulated from VA and with a non‐wear of at least 90 consecutive minutes of zero counts
VM60:	Days with SED, LPA and MVPA accumulated from VM and with a non‐wear time of at least 60 consecutive minutes of zero counts
VM90:	Days with SED, LPA and MVPA accumulated from VM and with a non‐wear time of at least 90 consecutive minutes of zero counts

Abbreviations: LPA, light‐intensity physical activity; MVPA, moderate‐to‐vigorous physical activity; SED, sedentary behaviours; VA, vertical/unilateral accelerometer data; VM, triaxial accelerometer data.

^a^ ActiLife software version 6.13.4 (ActiGraph LLC).

To further validate settings made in the data processing phase the four data sets with days of monitored data were transformed to data sets of first (i) valid or not valid days, then (ii) valid or not valid periods. To classify a day as valid, a minimum of either 300, 500 or 600 min/day was required to be monitored. Then to classify a period as valid, every consecutive period of 7 days that had been monitored required a minimum of either 3 or 4 valid days to be considered a valid period.

Finally, in the last step of evaluating settings made in data processing phase, SED, LPA and MVPA in valid days for valid periods were compared using the different settings in VA versus VM, non‐wear time and settings to classify a day and period as valid. The entire analytical procedure to evaluate settings made in the data processing phase is illustrated in Figure 1.

Figure 1Flowchart illustrating decisions made in the data processing phase.

**Figure:**
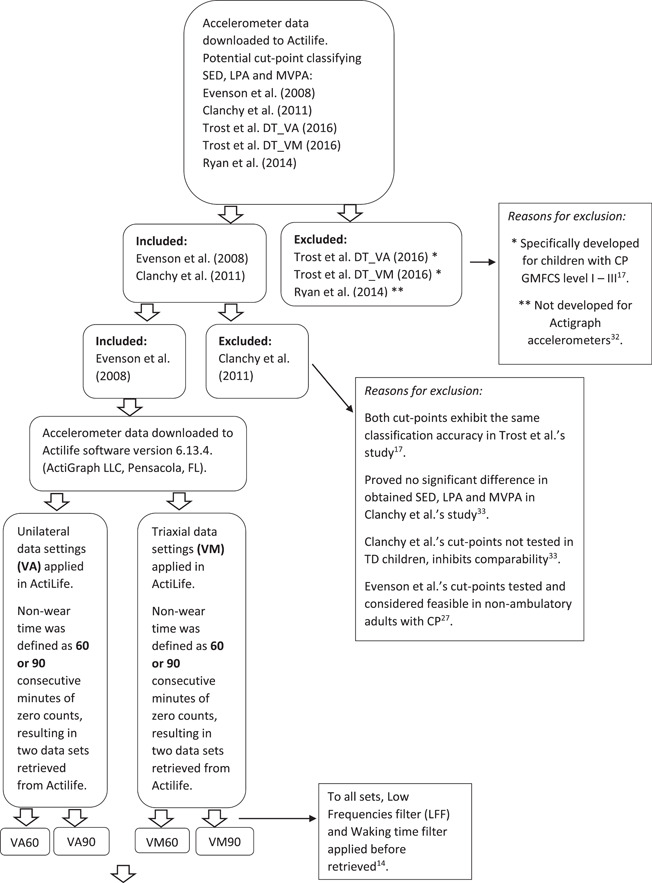

**Figure:**
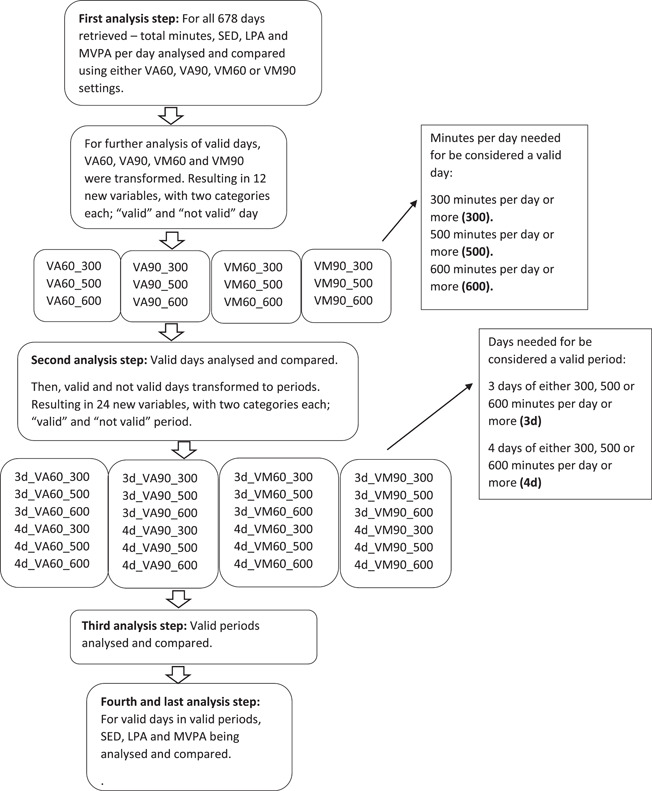

### 2.4 Statistical analysis

Data were tested for normal distribution and non‐parametric tests were used to test statistical significance. To analyse the difference in minutes per day obtained in total, in SED, in LPA and in MVPA when using VA versus VM and 60 versus 90 consecutive minutes of non‐wear time, a Friedman test with a post hoc Wilcoxon signed‐rank test was used. To analyse the difference in (i) valid days and (ii) valid periods, using the different settings to classify a day and period as valid, Cochran's *Q* test with a post hoc pairwise comparisons using Dunn's procedure with a Bonferroni correction for multiple comparisons was used. Lastly, to determine if there was a statistical difference in SED, LPA and MVPA for valid days in valid periods using VA or VM and 60 or 90 consecutive minutes of non‐wear time, a Kruskal–Wallis *H* test with a post hoc pairwise comparisons using Dunn's procedure with a Bonferroni correction for multiple comparisons was used (Kirkwood & Sterne, 2003; Pallant, 2020). For all analyses IBM SPSS Statistics version 26 was used.

## 3 RESULTS

### 3.1 Total minutes, total minutes in SED, LPA and MVPA recorded per day

In total, 678 days and 97 time periods were monitored. There was an overall statistically significant difference in minutes per day obtained in total, in SED, in LPA and in MVPA per day depending on which settings in VA versus VM and in non‐wear time that had been chosen: total time *χ* ^2^(2) = 1377.86, *p* < 0.001, SED *χ* ^2^(2) = 1387.96, *p* < 0.001, LPA *χ* ^2^(2) = 1384.51, *p* < 0.001 and MVPA *χ* ^2^(2) = 313.35, *p* < 0.001. For pairwise comparisons, there was a statistically significant reduction of minutes recorded if choosing VA instead of VM and a non‐wear time of 60 consecutive minutes instead of 90 min (Table 2).

**Table 2:** Pairwise comparisons for analysing statistically significant differences in median minutes obtained in total, in SED, LPA and MVPA, when using either VA or VM and either 60 or 90 consecutive minutes of non‐wear time for all days with data monitored

Pairwise comparisons	*Z*	*p* Value
Total minutes		
60VA–90VA	−19.51	*p* < 0.0125
60VM–90VM	−17.23	*p* < 0.0125
60VA–60VM	−21.44	*p* < 0.0125
90VA–90VM	−20.56	*p* < 0.0125
SED		
60VA_SED–90VA_SED	−19.46	*p* < 0.0125
60VM_SED–90VM_SED	−17.26	*p* < 0.0125
60VA_SED–60VM_SED	−21.36	*p* < 0.0125
90VA_SED–90VM_SED	−20.55	*p* < 0.0125
LPA		
60VA_LPA–90VA_LPA	−19.36	*p* < 0.0125
60VM_LPA–90VM_LPA	−14.89	*p* < 0.0125
60VA_LPA–60VM_LPA	−21.06	*p* < 0.0125
90VA_LPA–90VM_LPA	−20.01	*p* < 0.0125
MVPA		
60VA_MVPA–90VA_MVPA	−8.85	*p* < 0.0125
60VM_MVPA–90VM_MVPA	−6.56	*p* < 0.0125
60VA_MVPA–60VM_MVPA	−11.20	*p* < 0.0125
90VA_MVPA–90VM_MVPA	−8.93	*p* < 0.0125

Abbreviations: LPA, light‐intensity physical activity; MVPA, moderate‐to‐vigorous physical activity; SED, sedentary behaviours; VA, vertical/unilateral accelerometer data; VM, triaxial accelerometer data; *Z*, effect size; 60, 60 consecutive minutes of zero counts or more; 90, 90 consecutive minutes of zero counts or more.

### 3.2 Valid days and periods

There was an overall statistically significant difference in valid days recorded using different settings in VA versus VM, non‐wear time and minutes needed to classify a day as valid *χ* ^2^(2) 2477.45, *p* < 0.001. All pairwise combinations but VM90 compared to VM60 with at least 300 min recorded classifying a day as valid were proven to be statistically significantly different, *p* < 0.001. More specifically, statistically significantly more valid days were obtained when using VM rather than VA, non‐wear time was defined as 90 consecutive minutes of no registered counts instead of 60 min, and fewer minutes needed to classify a day as valid.

Similar trends for valid periods as for valid days were observed. More valid periods were obtained when using VM rather than VA, non‐wear time was defined as 90 consecutive minutes of zero counts instead of 60 min, and fewer minutes were needed to classify a day as valid days. Further, valid periods declined when 4 days of either 300, 500 or 600 min/day or more were needed to validate a period compared to 3 days.

There was an overall statistically significant difference in valid periods recorded using the different settings *χ* ^2^(2) 908.43, *p* < 0.001. Pairwise comparisons and statistically significant differences in valid periods are shown in Table 3.

**Table 3:** Pairwise comparisons for analysing statistically significant differences in valid periods when using either VA or VM and either 60 or 90 consecutive minutes of non‐wear time

Pairwise comparison made	300 Adj. Sig.^a^	500 Adj. Sig.^a^	600 Adj. Sig.^a^
3d 60VA–3d 60VM	0.07	**<0.001**	**<0.001**
3d 90VA–3d 90VM	1.00	**<0.001**	**<0.001**
3d 60VA–3d 90VA	1.00	**0.007**	0.07
3d 60VM–3d 90VM	1.00	1.00	1.00
4d 60VA–4d 60VM	**0.003**	**<0.001**	**<0.001**
4d 90VA–4d 90VM	0.95	**<0.001**	**<0.001**
4d 60VA–4d 90VA	1.00	**0.007**	1.00
4d 60VM–4d 90VM	1.00	1.00	1.00
3d 60VA–4d 60VA	1.00	1.00	1.00
3d 90VA–4d 90VA	1.00	1.00	1.00
3d 60VM–4d 60VM	1.00	1.00	1.00
3d 90VM–4d 90VM	1.00	1.00	1.00

*Note*: For pairwise comparison, each row tests the null hypothesis that Sample 1 and Sample 2 are the same.

Asymptotic significances (two‐sided tests are displayed). The significance level is 0.05. Bold values show significant differences.

Abbreviations: VA, vertical/unilateral accelerometer data; VM, triaxial accelerometer data; 60, 60 consecutive minutes of zero counts or more; 90, 90 consecutive minutes of zero counts or more; 300, 500 or 600, number of minutes of recorded accelerometer data that were needed for it to be considered a valid day;  3d or 4d, number of valid days needed for the period to be considered as valid.

^a^ Significance values have been adjusted by the Bonferroni correction for multiple tests.

### 3.3 SED, LPA and MVPA for valid days in valid periods

Median minutes in SED increased when using VM and non‐wear time of 90 consecutive minutes. For LPA and MVPA median minutes increased when using VA and non‐wear time of 60 consecutive minutes. Least median minutes for LPA and MVPA were obtained when using VM and non‐wear time of 90 consecutive minutes (Table 4).

**Table 4:** Number of days obtained, and median minutes and (IQR) monitored for SED, LPA or MVPA for valid days in valid periods, given different settings in VA or VM, non‐wear times, recorded minutes needed for classifying a valid day and number of days needed for a period

	*N*	300 SED	300 LPA	300 MVPA	*N*	500 SED	500 LPA	500 MVPA	*N*	600 SED	600 LPA	600 MVPA
3d 60VA	363	440 (353.33–562.17)	51 (34.33–83)	1.67 (0.83–3.83)	151	576.33 (492.67–659)	77.17 (57.33–104.6)	2.5 (1.33–5.17)	86	616.92 (575.5–694.58)	84.42 (68.13–108.04)	3 (1.5–5.71)
3d 60VM	551	591 (465.17–686.17)	38.5 (23.00–67.83)	1.5 (0.83–3)	417	626.83 (554.67–714.33)	46.5 (28.83–76.92)	1.5 (0.83–3.17)	294	675.08 (606.83–740.5)	53.92 (35.36–85.25)	1.67 (0.83–3.5)
3d 90VA	459	526.17 (401.17–641,04)	46.17 (28.25–74.96)	1.67 (0.83–3.46)	285	605.83 (533.67–683.67)	57.67 (39.17–88.67)	1.83 (1–4.17)	174	658.5 (592.5–719.25)	68.17 (49.29–91.13)	2.17 (1.17–4.21)
3d 90VM	578	646.67 (527.08–732.12)	37.5 (21.67–65.91)	1.5 (0.83–3)	499	669.67 (581.33–747.33)	40.17 (24.17–70.83)	1.5 (0.83–3)	392	698.75 (633.75–758.29)	46.41 (28.17–75.04)	1.5 (0.83–3)
4d 60VA	342	441.08 (353.46–571.86)	53.42 (36.25–84.83)	1.83 (0.83–4)	125	584 (511.42–666.17)	79.33 (60.67–104)	2.5 (1.5–5,17)	71	622.5 (579–696.33)	84.83 (66.5–105.33)	2.5 (1.5–5.67)
4d 60VM	548	591.08 (466.17–686.92)	38.67 (23.17–67.96)	1.5 (0.83–3)	384	639.42 (559.67–719.63)	48.33 (29.54–8.96)	1.58 (0.83–3.17)	261	679.33 (612.08–741.42)	54.83 (36.08–85.33)	1.67 (0.83–3.33)
4d 90VA	439	538.67 (420.5–646.5)	46.67 (29.83–76.17)	1.83 (0.83–3.5)	261	609.33 (537.83–689.5)	57.33 (38.92–86.5)	1.83 (0.83–4.17)	138	666 (609.21–721.5)	72.08 (50.21–91.67)	2.25 (1.29–5)
4d 90VM	575	648.17 (531.5–734.5)	37.67 (22–66.17)	1.5 (0.83–3)	475	673 (583–748.83)	41.5 (25.17–72.33)	1.5 (0.83–3)	371	699.17 (633.33–760)	47 (28.67–75.67)	1.5 (0.83–3)

Abbreviations: IQR, interquartile range; LPA, light‐intensity physical activity; MVPA, moderate‐to‐vigorous physical activity; SED, sedentary behaviours; VA, vertical/unilateral accelerometer data; VM, triaxial accelerometer/vector magnitude data; 60, non‐wear time of 60 consecutive minutes of zero counts or more; 90, non‐wear time of 90 consecutive minutes of zero count or mores; 300, 500 or 600, number of minutes of recorded accelerometer data that were needed for it to be considered a valid day.

Variables with different criteria of how to classify a valid day were analysed separately. Pairwise comparisons and statistically significant differences in valid periods are shown in Table 5.

**Table 5:** Pairwise comparisons for analysing statistically significant differences in median time for SED, LPA or MVPA when comparing VA and VM and different non‐wear time, in valid days in valid periods

Pairwise comparisons made	300 SED Adj. Sig.^a^	300 LPA Adj. Sig.^a^	300 MVPA Adj. Sig.^a^	500 SED Adj. Sig.^a^	500 LPA Adj. Sig.^a^	500 MVPA Adj. Sig.^a^	600 SED Adj. Sig.^a^	600 LPA Adj. Sig.^a^	600 MVPA Adj. Sig.^a^
3d 60VA–3d_60VM	**<0.001**	**<0.001**	0.394	**<0.001**	**<0.001**	**0.003**	**0.001**	**<0.001**	**<0.001**
3d_90VA–3d_90VM	**<0.001**	**0.001**	1.00	**<0.001**	**<0.001**	**0.037**	**<0.001**	**<0.001**	0.094
3d_60VA–3d_90VA	**<0.001**	0.44	1.00	0.331	**<0.001**	1.00	0.3	**0.022**	0.257
3d_60VM–3d_90VM	**<0.001**	1.00	1.00	**0.001**	0.529	1.00	**0.022**	**0.026**	1.00
4d_60VA–4d_60VM	**<0.001**	**<0.001**	1.00	**0.001**	**<0.001**	**0.002**	**0.007**	**<0.001**	**0.002**
4d_90VA–4d_90VM	**<0.001**	**<0.001**	0.152	1.00	**<0.001**	0.598	**0.020**	**<0.001**	0.111
4d_60VA–4d_90VA	**<0.001**	0.055	0.412	**0.013**	0.522	1.00	0.314	0.403	1.00
4d_60VM–4d_90VM	**<0.001**	1.00	1.00	**<0.001**	**<0.001**	0.412	**0.025**	0.54	1.00
3d_60VA–4d_60VA	1.00	1.00	1.00	1.00	1.00	1.00	1.00	1.00	1.00
3d_60VM–4d_60VM	1.00	1.00	1.00	1.00	1.00	1.00	1.00	1.00	1.00
3d_90VA–4d_90VA	1.00	1.00	1.00	1.00	1.00	1.00	1.00	1.00	1.00
3d_90VM–4d_90VM	1.00	1.00	1.00	1.00	1.00	1.00	1.00	1.00	1.00

*Note*: For pairwise comparison, each row tests the null hypothesis that Sample 1 and Sample 2 are the same. Asymptotic significances (2‐sided tests are displayed). The significance level is 0.05. Bold values show significant differences.

Abbreviations: LPA, light‐intensity physical activity; MVPA, moderate‐to‐vigorous physical activity; SED, sedentary behaviours; VA, vertical/unilateral accelerometer data; VM, triaxial accelerometer data; 60, 60 consecutive minutes of zero counts or more; 90, 90 consecutive minutes of zero counts or more; 300, 500 or 600, number of minutes of recorded accelerometer data that were needed for it to be considered a valid day; 3d or 4d, number of valid days that were needed for the period to be considered valid.

^a^ Significance values have been adjusted by the Bonferroni correction for multiple tests.

## 4 DISCUSSION

This is to our knowledge the first study evaluating settings in the data processing phase for non‐ambulant children and adolescents with CP, thus enabling standardized data processing settings to be used when assessing PA by ActiGraph GT3X accelerometer in non‐ambulant children and adolescents with CP in future research and clinical settings.

### 4.1 VA versus VM

Although VM is commonly used when describing PA in children and adolescents nowadays (Migueles et al., 2017; Romanzini et al., 2014; Steene‐Johannessen et al., 2020), there are conflicting results comparing SED, LPA and MVPA retrieved from VA or VM in the literature. In Romanzini et al. and Trost et al.'s studies validating accelerometer cut‐points in children and adolescents with either no physical disability or ambulant children and adolescents with CP, GMFCS level I–III, no statistically significant differences in classification accuracy between VA and VM were obtained (Trots et al., 2016; Romanzini et al., 2014). Thus, the same amount of SED, LPA and MVPA was retrieved. However, in both studies the physical activities performed by the study participants contained a lot of vertical movements, which may explain the similar results between VA and VM (Trots et al., 2016; Romanzini et al., 2014). Non‐ambulant children and adolescents with CP are unable to sit or walk without support and need assistance to be physically active, which restricts their abilities to perform vertical movements (Palisano et al., 1997). Restricted abilities to perform vertical movements may explain the statistically significant difference in median minutes recorded for each intensity level (SED, LPA and MVPA) when comparing VA with VM for all days recorded in our study.

Further, the statistically significant difference in VA versus VM in our study is further strengthened by Oftedal et al.'s study. When Oftedal et al. (2014) validated cut‐points to classify SED from PA in ambulant and nonambulant toddlers with CP, GMFCS levels I–V, VA was found to overestimate SED in non‐ambulant toddlers, and so VA was not recommended. Interestingly, in our study VA produced statistically significantly fewer median minutes in SED when analysing both all days recorded and valid days in valid periods. Even though there was a statistically significant difference in median time in both SED, LPA and MVPA for all days recorded, the most significant difference in median was obtained when comparing SED accumulated from VA or VM. For example, a median of 303.17  min/day of SED was reported for VA60 compared with 610.42 min/day of SED when using VM90. Fewer minutes of SED resulted in statistically significantly fewer valid days and periods possible to use in further analysis, regardless of the criteria set to determine a valid day.

Although the results of SED in our study and Oftedal et al.'s study differ, both studies indicate less activity counts being accumulated by VA. Hence, VM appears to estimate SED more precisely in non‐ambulant children and adolescents with CP.

### 4.2 Different non‐wear time

As well as comparing VA and VM, a comparison of 60 consecutive minutes of non‐wear time versus at least 90 consecutive minutes was made. A non‐wear time of 60 consecutive minutes of zero counts or more was chosen as it is commonly recommended and used in children and adolescents (Aadland et al., 2018; Chinapaw et al., 2014; Choi et al., 2011). As increasing knowledge of SED in children and adolescents is recognized, though, longer periods of non‐wear time have been applied in subgroups of children and adolescents being less physically active, commonly using 90 consecutive minutes of zero counts as the recommended non‐wear time (Janssen et al., 2015; Toftager et al., 2013).

Median time in total time, SED, LPA and MVPA statistically significantly increased further when using a non‐wear time set to 90 consecutive minutes of zero count instead of 60 min when all days were analysed. However, when analysing periods of either 3 or 4 days, there was no statistically significant difference in 60VM compared to 90VM, regardless of whether 3 or 4 days was chosen. Fewer statistically significant differences between non‐wear time of 60 and 90 consecutive minutes were observed for SED, LPA and MVPA for valid days in valid periods. Fewer statistically significant differences in the latter analytical tests may indicate less of an impact of different non‐wear times used compared to VA and VM in non‐ambulant children and adolescents with CP. Nevertheless, considering non‐ambulant children and adolescents with CP have difficulties in moving (Rosenbaum et al., 2007; Palinsano et al., 1997), a non‐wear time in line with other sedentary subgroups of children and adolescents seems more applicable (Janssen et al., 2015; Toftager et al., 2013).

### 4.3 Valid days and periods

When evaluating settings made to classify a day as valid, three settings were chosen. The minimum of 300 min monitored per day has previously been used to classify a day as valid in populations of children, adolescents and adults with CP (Claridge et al., 2015; Gorter et al., 2012; Strath et al., 2012), whereas at least 500 min/day is commonly used among children and adolescents (Cooper et al., 2015; Trost et al., 2005) and 600 min is used and recommended to validate a day in children, adolescents, but most commonly adults (Migueles et al., 2017). Although a day with the minimum of 300 min/day needed to classify a day as valid produced statistically significantly more valid days, from a clinical and research perspective at least 500 or 600 min monitored per day generate a greater understanding of how the child's or adolescent's PA looks (Cooper et al., 2015; Migueles et al., 2017; Trost et al., 2005).

Whereas 499 valid days (73.6%) were obtained when using at least 500 min/day to classify a day as valid, 413 days (60.9%) were obtained when 600 min was set. As there was a statistically significant difference between 500 and 600  min, with 500 min generating more days to analyse, the minimum of 500 min/day to classify a day as valid may be considered the criterion.

No statistically significant differences in periods were found when using 3 or 4 days of either 300, 500 or 600 min or more recorded per day set as a criterion. Even though at least four valid days are commonly recommended for a period to be considered as valid (Migueles et al., 2017; Strath et al., 2012), we found no statistically significant differences in periods of 3 or 4 days when all other settings were the same. This is in line with Mitchelle et al.'s study, which tested reliability when using Evenson et al.'s cut‐points in ambulatory children with CP. Mitchell et al. (2015) concluded that a minimum of 3 days was needed to achieve reliable results in SED, LPA and MVPA (Mitchell et al., 2015). Further, periods of fewer valid days have been proven in more sedentary groups (Arvidsson et al., 2019). Consequently, using at least three valid days for each period to assess SED, LPA and MVPA in non‐ambulant children and adolescents with CP might be recommended.

### 4.4 Methodological considerations

Nonparametric tests were chosen to evaluate settings made in the data processing phase as the assumption of normal distribution was violated. We did not remove outliers nor transform the data to potentially increase normal distribution. Non‐ambulant children and adolescents with CP are a very heterogeneous group with some children and adolescents being more physically active than others (Gulati & Sondhi, 2018; Rosenbaum et al., 2007). Hence, including outliers in the sample may potentially reflect the population better.

### 4.5 Strengths and limitations

For our study, Evenson et al. cut‐points were used. In 2020, Bianchim et al. reviewed accelerometer cut‐points set to determine PA intensity levels and SED in children and adolescents with chronical disabilities. For children and adolescents with CP there were five different sets of cut‐points. Four out of those were established for ActiGraph accelerometers (Bianchim et al., 2020). Although Evenson et al. (2008) cut‐points are established for VA, which could be considered a limitation of our study, they were considered the most applicable cut‐points to use. Evenson et al. cut‐points are the only ones tested in non‐ambulant children and adolescents with CP, as well as in children and adolescents without disabilities (Clanchy et al., 2011; Evenson et al., 2008; Gorter et al., 2012).

On the other hand, our results are strengthened by the large number of days we were able to analyse, which is a consequence of the large number of participants in this study. The number of participants in our study is larger than in previous studies examining Evenson et al.'s cut‐points or in studies investigating the relationship of PA and non‐ambulant children and adolescents with CP (Lai et al., 2021; Strath et al., 2012; Verschuren et al., 2016).

Lastly, calibration and validation of cut‐points and VA and VM, regardless of study population, is normally done against oxygen uptake and heart rate measurements (Clanchy et al., 2011; Evenson et al., 2008; Freedson et al., 1998; Migueles et al., 2017; Romanzini et al., 2014). Although we had a large number of days to analyse, the data were drawn from two already existing studies. Hence, we had no possibility to calibrate and validate cut‐points and VM or VA against oxygen uptake and heart rate measurements to further strengthen our results. As data had already been collected, we were also unable to use logbooks which could have further validated non‐wear times (Aadland et al., 2018).

### 4.6 Future studies

In future studies we recommend to further validate VA and VM against oxygen uptake and heart rate measurements, as well as non‐wear times against logbooks in non‐ambulant children and adolescents with CP. To increase the reliability of the chosen setting of how to classify a day and period as valid one could in future studies explore if the settings statistically obtain an intraclass correlation coefficient of 0.80 (Aadland & Ylvisåker, 2015; Mitchell et al., 2015). As well as evaluating settings made in the data processing phase, settings made before data collection, such as device placement, should be further evaluated.

## 5 CONCLUSION

By evaluating settings made in the data processing phase while using Evenson et al. cut‐points, standardized data processing settings have been validated. Advancements in what settings to use in the data processing phase will help to correctly assess the levels of PA and SED for non‐ambulant children and adolescents with CP. They will also help increase the reliability of comparisons between studies. Our study suggests using the settings VM and a non‐wear time of 90 constitutive minutes, 500 min recorded per day with periods of at least three valid days when assessing PA objectively by the ActiGraph GT3X accelerometer in non‐ambulant children and adolescents with CP.

## CONFLICT OF INTEREST

The authors declare no conflict of interest.

## Data Availability

The data used in this study contains sensitive information about the study participants and they did not provide consent for public data sharing. The current approval by the Regional Ethical Review Board in Lund, Sweden (2019‐00106) does not include data sharing. A minimal data set could be shared by request from a qualified academic investigator for the sole purpose of replicating the present study, provided the data transfer is in agreement with EU legislation on the general data protection regulation and approval by the Swedish Ethical Review Authority.
